# Primary Cutaneous Cryptococcosis in a Patient on Fingolimod: A Case Report

**DOI:** 10.7759/cureus.16444

**Published:** 2021-07-17

**Authors:** Deena Dahshan, Sofanit A Dessie, Jonathan Cuda, Elie Khalil

**Affiliations:** 1 Internal Medicine, Marshall University Joan C. Edwards School of Medicine, Huntington, USA; 2 Pathology, Marshall University Joan C. Edwards School of Medicine, Huntington, USA; 3 Infectious Diseases, Marshall University Joan C. Edwards School of Medicine, Huntington, USA

**Keywords:** multiple sclerosis, primary cutaneous cryptococcosis, fingolimod, infectious disease, immunosuppression

## Abstract

Primary cutaneous cryptococcosis is an uncommon condition. Patients with immunosuppression and those of older age are more susceptible to infection, warranting investigations into underlying systemic disease. We report the case of a 49-year-old male with multiple sclerosis in remission on fingolimod who presented with a non-healing skin lesion on his upper thigh for a duration of two years. Skin biopsy showed dermal parasitized histiocytes, and serum antigens for histoplasmosis and *Cryptococcus* were negative. Further investigation with polymerase chain reaction (PCR) demonstrated cutaneous cryptococcal infection, with no associated systemic signs or symptoms. This case report highlights an uncommon presentation of cutaneous cryptococcosis on an unexposed skin surface with successful and rapid improvement following fluconazole therapy without fingolimod discontinuation.

## Introduction

Cutaneous presentations of cryptococcal infections are typically associated with disseminated disease. The discovery of cutaneous cryptococcal infection in medically-induced immunosuppressed patients necessitates a discussion on screening to reduce mortality from untreated systemic infection. We present a case report where we analyze the clinical morphology and presentation of an isolated primary cutaneous cryptococcosis infection associated with fingolimod therapy for multiple sclerosis. A biopsy is essential for ambiguous skin lesions, especially in immunosuppression settings, to exclude such opportunistic infections. We believe that continued reporting of this complication associated with fingolimod therapy will raise greater awareness among clinicians to follow patients closely for skin changes throughout the course of this therapy and to consider the involvement of Infectious Diseases. Surveillance mechanisms for opportunistic infections with immune modulation in multiple sclerosis have not yet been clearly established, but a high index of suspicion should help to identify and manage high-risk patients appropriately and promptly.

This case report highlights the effectiveness of extended oral fluconazole therapy in treating primary cutaneous cryptococcosis without the need for intravenous antifungal regimens typically used in disseminated disease and further rapid resolution of the lesion without discontinuing fingolimod for multiple sclerosis. This reduces potential multiple sclerosis-related adverse effects. Notably, prior to deciding on a definitive therapy regimen and mode of delivery, clinical and radiographic exclusion of a disseminated infection is of utmost importance. While the cutaneous cryptococcal infection is known to be associated with disseminated disease in immunocompromised patients, there are increasing reports concerning primary cutaneous cryptococcosis infection associated with fingolimod immunosuppressive therapy on exposed skin [1,2]. This is the first report of a case involving an unexposed skin surface and effective resolution with continued fingolimod therapy for multiple sclerosis.

## Case presentation

**Figure 1:**
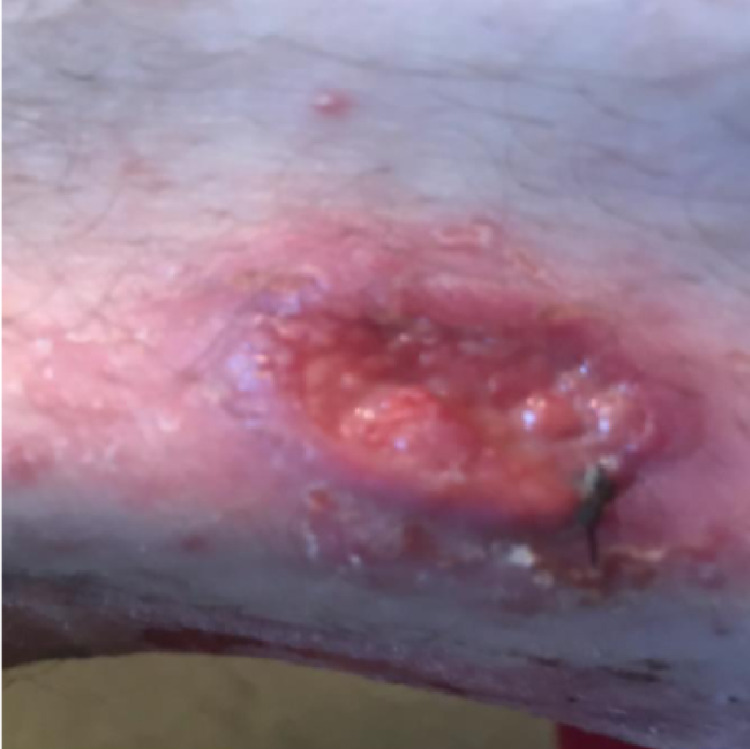
Picture of the skin lesion found on the left thigh on initial presentation

A 49-year-old male, with a medical history of multiple sclerosis in remission on fingolimod monotherapy for nine years, presented in an outpatient clinic for a non-healing lesion on the upper thigh. He had noticed the skin lesion two years before presenting to dermatology and had been previously treated with multiple rounds of oral antibiotics and over-the-counter topical ointments, which had resulted in no improvement or change in the size of the lesion. The patient had no recollection of trauma to the area. He denied itching, swelling, redness, or pain associated with the lesion. He also denied fever, night sweats, malaise, weight loss, headache, and other neurological and systemic symptoms. Physical exam was only remarkable for a 2-cm, round, ulcerated, cutaneous nodule on the left upper thigh with no significant surrounding erythema, discharge, tenderness, or necrosis (Figure 1). There were no other similar skin lesions on his body.

**Figure 2:**
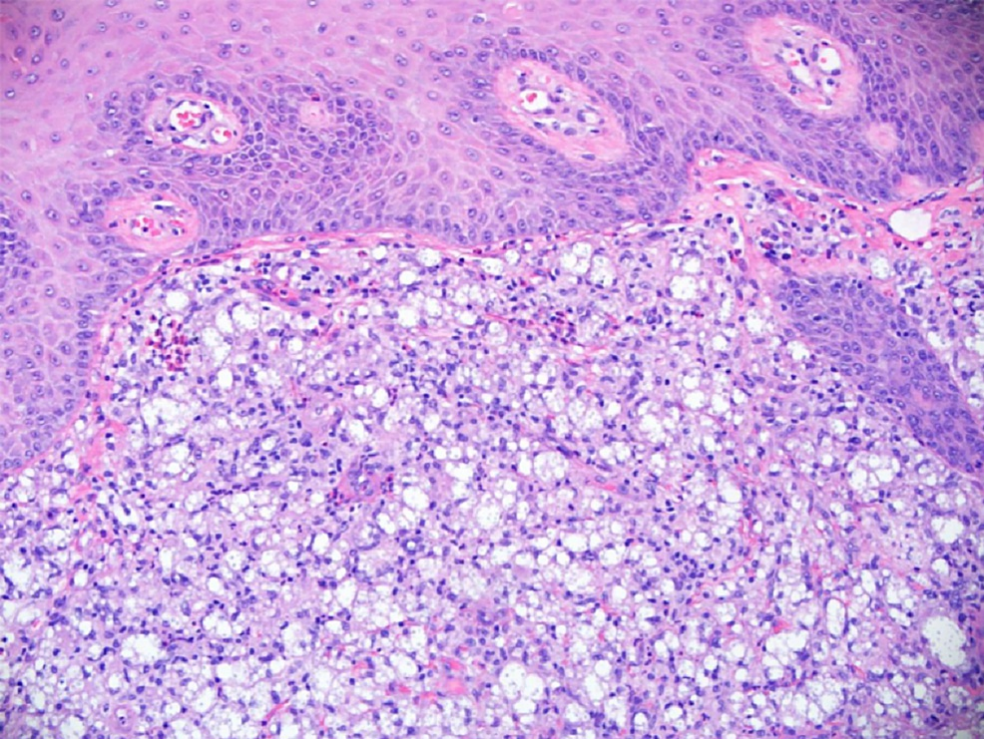
Punch biopsy of the skin lesion showing numerous yeast forms predominantly within the cytoplasm of macrophages

Laboratory results revealed a low lymphocyte count of 6.2%, but an unremarkable white cell count of 5.6 x 10^9^/L. HIV serology was negative. The absolute lymphocyte count was 0.3 x 10^9^/L. Punch biopsy of the skin lesion showed a deep fungal infection with numerous yeast forms predominantly within the cytoplasm of macrophages (Figure 2).

**Figure 3:**
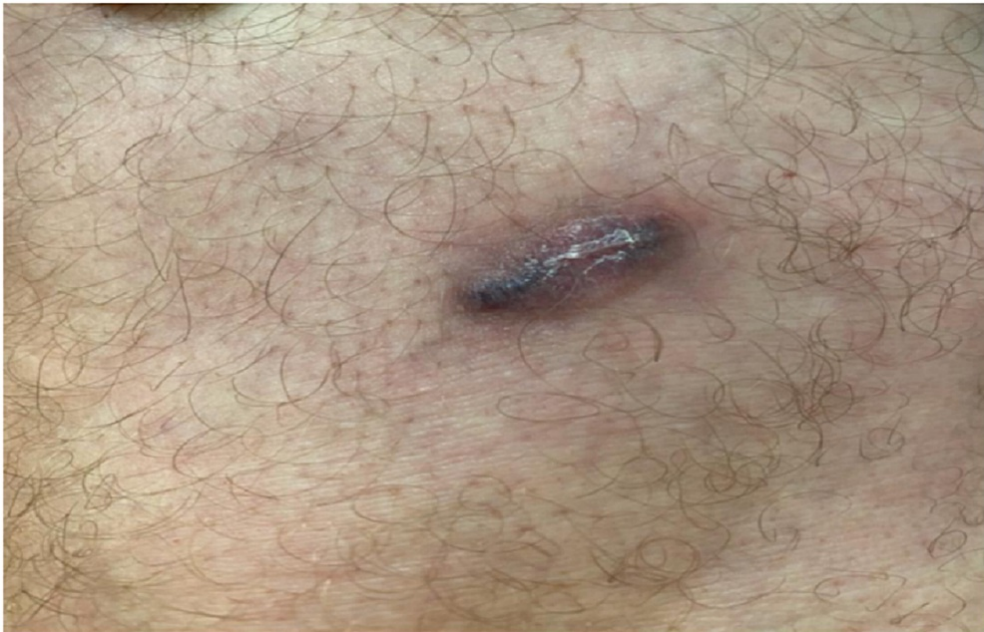
Picture of healing and improvement in the size of the skin lesion after four-month treatment with oral fluconazole

The differential diagnosis included both histoplasmosis and cryptococcosis. Histoplasmosis was prominently considered given the geographical epidemiology, and the Infectious Disease was consulted. Histoplasmosis serology and antigens were negative. Serum was also negative for cryptococcal antigens, and there was no evidence of disseminated fungal infection. Attempts at tissue culture were unsuccessful. The paraffin block was sent for fungal polymerase chain reaction (PCR) and was found to be positive for *Cryptococcus neoformans*. There were no clinical indications for a lumbar puncture given the indolent course and absence of neurological symptoms. The patient was started on oral fluconazole 600 mg twice a day for 14 days followed by 400 mg twice a day for four months. This resulted in significant rapid improvement within the first two weeks with complete healing of the skin lesion at the end of the four-month treatment (Figure 3).

The patient tolerated the treatment well without discontinuation of his fingolimod regimen for multiple sclerosis. Liver and kidney functions were monitored throughout the course of treatment and were normal. The patient was advised to report any new lesions or recurrences after completing the four-month course of fluconazole. There was no recurrence at the one-year follow-up.

## Discussion

*Cryptococcus neoformans* is an invasive fungus found in soil and pigeon droppings, causing infection in susceptible immunocompromised patients. Extrapulmonary cryptococcal infection is an AIDS-defining illness. The most common acquired mechanism of infection is lung inhalation, with numerous cases of pulmonary infection in patients on disease-modifying therapy [3]. Skin infection is usually secondary and must be comprehensively investigated to rule out disseminated disease [4].

Cutaneous cryptococcal disease is rarely an isolated finding and is typically the result of primary inoculation [5]. Lesions mostly present on exposed areas of skin [6]. It usually occurs as a single lesion that does not extend beyond subcutaneous tissue without evidence of systemic dissemination. In immunocompromised patients, *Cryptococcus* can cause disseminated meningitis. This typically presents as a subacute meningoencephalitis with neurological symptoms of headache, altered mental status, lethargy, fever, and stiff neck between 6-12 weeks of the onset of presentation [7]. Patients who are positive for HIV may have nonspecific symptoms [7]. While our patient was on fingolimod, he was not positive for HIV. His lesion had been present for two years prior to the presentation with an indolent course and he did not have any signs or symptoms indicating underlying systemic or neurologic disease that would have warranted a spinal tap.

Our patient's case, with a lesion on the upper thigh, was a unique presentation compared to previous cases reported involving the scalp and forehead [1,2]. To our knowledge, there are only two other reported cases of primary cutaneous cryptococcosis with associated fingolimod therapy (Table 1) [1,2]. Primary cutaneous cryptococcal infections may present with chancriform syndromes, involving an ulcerative lesion at the primary site of inoculation and regional lymphadenopathy [8]. Other manifestations may include ulceration, cellulitis, or whitlow-like lesions [6]. Early diagnosis can be challenging, and sources of immunosuppression should be investigated. Previous cases have involved older patients, with a history of trauma and associated pain at the lesion site (Table 1) [1,2].

**Table 1:** Literature review of primary cutaneous cryptococcosis with fingolimod therapy

Author	Patient presentation	Fingolimod duration	Management
Patil et al., 2020 [1]	63-year-old male with a 4-year history of 3 non-healing scalp lesions, low CD4 count (13 cells)	7 years	6 months of fluconazole 400 mg daily; sulfamethoxazole/trimethoprim prophylaxis for pneumocystis. Fingolimod discontinued
Forrestel et al., 2016 [2]	62-year-old female with a painful 3-week nodule on the forehead with a history of trauma 6 months prior	3 years	6 weeks of fluconazole 800 mg loading dose followed by 400 mg daily. Fingolimod discontinued

The cryptococcal pathogen is unique in its immune escape mechanisms with latent infection and potential reactivation, manifesting in susceptible immunocompromised patients [9]. The most commonly reported therapy-related immunosuppression risk factor for primary cutaneous cryptococcal infection is systemic corticosteroid use [10]. Previous literature describes age between 50-60s as a critical risk factor due to natural immunosenescence with further increased risk with long-term use of fingolimod potentiating immunosenescence-like changes in T cell subsets [9]. Fingolimod is the first oral disease-modifying option for relapsing multiple sclerosis since 2010, an effective alternative to injection disease-modifying therapy [11]. Natalizumab and fingolimod are the only multiple sclerosis therapies that are complicated by cryptococcal infection to date [1]. Our patient did not have a history of corticosteroid use and his identified associated risk factor for infection was his nine-year history of fingolimod therapy.

Our patient was successfully cured with a four-month course of oral fluconazole therapy. While complicated by *Cryptococcus* infection on fingolimod therapy, our patient was effectively managed without fingolimod discontinuation. This decision was made based on the patient’s rapid improvement after the first two weeks of fluconazole. However, it was decided that fingolimod would be held if there was a poor response to treatment or worsening of the lesion. The patient’s lesion resolved following fluconazole treatment with no recurrence at one-year follow-up, indicating successful management without disruption of fingolimod for his multiple sclerosis. While this is unique compared to previous cases of primary cutaneous *Cryptococcus* infections in patients with multiple sclerosis on fingolimod, this individualized management reduced any potential flare-up of our patient’s multiple sclerosis.

A literature review detailing successful treatment of primary cutaneous cryptococcal infections in patients with underlying immunocompromise includes surgical excision and medical management with fluconazole therapy, 5-flucytosine, or amphotericin B [10]. With regard to the two previously reported cases of primary cutaneous cryptococcosis in patients on fingolimod, six months of fluconazole 400 mg daily was used in one case, while six weeks of fluconazole 800 mg loading dose followed by 400 mg daily was used in the other [1,2]. Fluconazole interferes with fungal cell membrane synthesis as it inhibits the synthesis of ergosterol and targets fungal cytochrome P450 enzyme activity. Fluconazole treatment is not without risk of adverse reactions and monitoring the patients closely allows for appropriate adjustment and intervention when necessary. For the duration of this therapy, patients should be monitored for baseline creatinine for pharmacokinetics, liver function tests for hepatotoxicity, EKG changes for QT prolongation, as well as for interactions with other medications.

Cutaneous presentations of *Cryptococcus* necessitate discussion on recognizing opportunistic infections to reduce mortality from underlying systemic disease. This case report highlights a rare presentation of primary cutaneous cryptococcosis in a patient on fingolimod with effective management following incidental recognition. Independent of age, disease-modifying agents for patients with multiple sclerosis may increase susceptibility to infection, and clinicians should preemptively counsel patients and be aware of the risks. To our knowledge, only two other cases of primary cutaneous cryptococcosis with associated fingolimod use have been previously reported, involving exposed skin surfaces in older patients. This case demonstrates successful management with fluconazole without the discontinuation of fingolimod therapy for our patient's multiple sclerosis.

## Conclusions

We discussed a case involving a rare presentation of primary cutaneous cryptococcosis in a patient on fingolimod with effective management following diagnostic evaluation. This case report adds to the scarce body of literature on primary cutaneous cryptococcosis with associated fingolimod use, as only two other similar cases have been previously reported, involving exposed skin surfaces in older patients. The use of disease-modifying agents in patients with multiple sclerosis may render them more susceptible to infection independent of age, and clinicians should preemptively counsel patients and be aware of the risks. This case illustrates a favorable patient outcome following treatment with fluconazole without the discontinuation of fingolimod therapy for our patient's multiple sclerosis.
